# Supplementary material for: Hairpin protein partitioning from the ER to lipid droplets involves major structural rearrangements
Source: Nat Commun. 2024 May 27;15:4504. doi: 10.1038/s41467-024-48843-8 (PMC11130287; doi:10.1038/s41467-024-48843-8)
Supplement: Supplementary file 1 — Supplementary Information [file 41467_2024_48843_MOESM1_ESM.pdf]

Hairpin protein partitioning from the ER to lipid droplets involves major structural rearrangements

Ravi Dhiman[1,6], Rehani S. Perera[1,6], Chetan S. Poojari[2,6], Haakon T. A. Wiedemann[3], Reinhard Kappl[4], Christopher W. M. Kay[3,5], Jochen S. Hub[2], Bianca Schrul[1]*

[1] Medical Biochemistry and Molecular Biology, Center for Molecular Signaling (PZMS), Faculty of Medicine, Saarland University, 66421 Homburg/Saar, Germany

[2] Theoretical Physics and Center for Biophysics, Saarland University, 66123 Saarbrücken, Germany.

[3] Physical Chemistry and Chemistry Education, Saarland University, 66123 Saarbrücken, Germany.

[4] Department of Biophysics, Center for Integrative Physiology and Molecular Medicine (CIPMM), Faculty of Medicine, Saarland University, 66421 Homburg/Saar, Germany

[5] London Centre for Nanotechnology, University College London, London, WC1H 0AH, United Kingdom

[6] These authors contributed equally: Ravi Dhiman, Rehani S. Perera, Chetan S. Poojari

[*] corresponding author: bianca.schrul@uks.eu

SUPPLEMENTARY INFORMATION

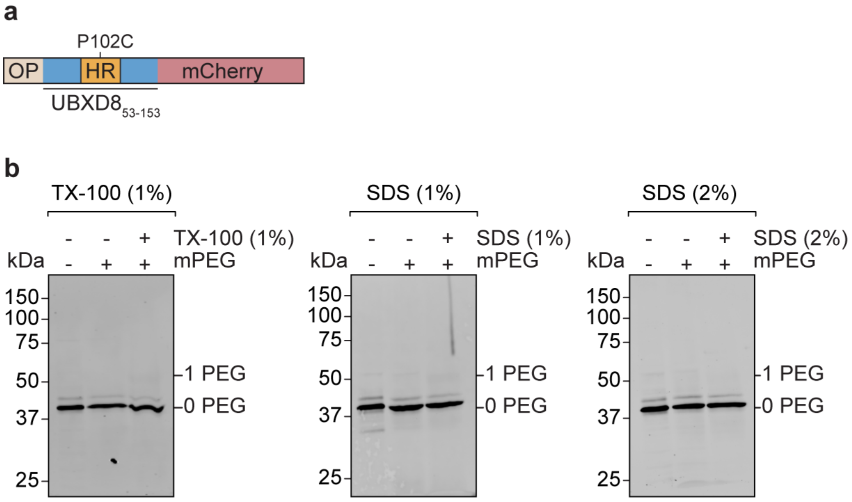

Dhiman *et al*., Supplementary Fig. 1

Supplementary Fig. 1:

P102C in opUBXD8$_{53\text{-}153}$mCherry is inaccessible to PEGylation under different detergent solubilization conditions

(a) Schematic outline of opUBXD8$_{53\text{-}153}$mCherry with single cysteine substitution at position P102. **(b)** Immunoblots using anti mCherry antibodies showing the PEGylation data for the opUBXD8$_{53\text{-}153}$mCherry single cysteine mutant P102C in ER bilayer membranes upon different solubilization conditions. First lanes: negative controls without the addition of mPEG, second lanes: samples treated with mPEG, third lanes: samples were solubilized with 1% Triton X-100 (left), 1% SDS (middle) or 2% SDS (right) before subjection to mPEG.

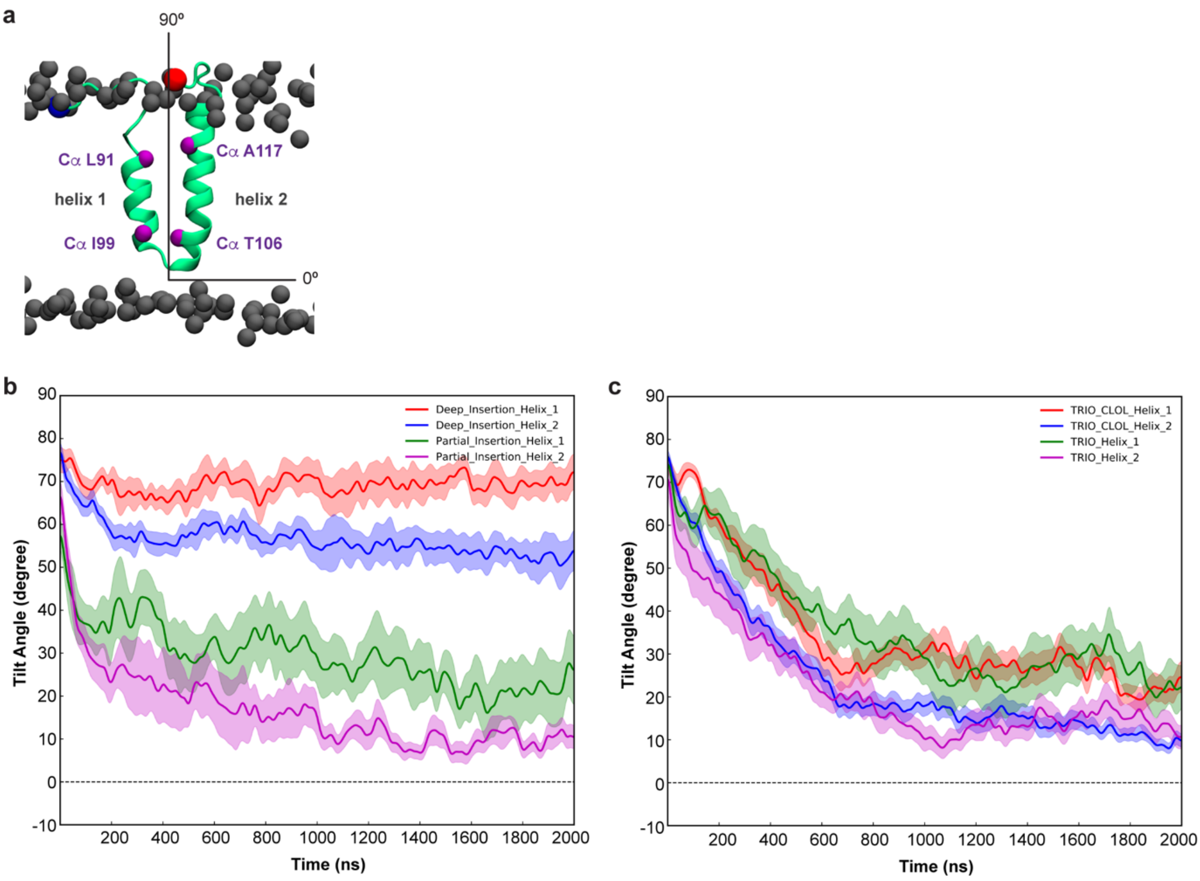

Dhiman *et al.*, Supplementary Fig. 2

Supplementary Fig. 2:

UBXD8$_{80\text{-}128}$ tilt angle measurements

(a) Illustration of tilt angle measurements: To define the helix tilt angle of UBXD8$_{80\text{-}128}$ relative to the membrane surface, we used the Cα–Cα vector between positions L91 and I99 for the first helix and between residues T106 and A117 in the second helix. **(b+c)** Tilt angles over the simulation time for UBXD8$_{80\text{-}128}$ either deeply inserted or partially inserted into a POPC bilayer as indicated (b) or inserted into either POPC-triolein/cholesteryl-oleate-POPC or POPC-triolein-POPC trilayer systems mimicking the LD monolayer membrane as indicated. Source data are provided as a source data file. Lines and shaded areas show mean and +/- SEM, respectively (n=5simulations).

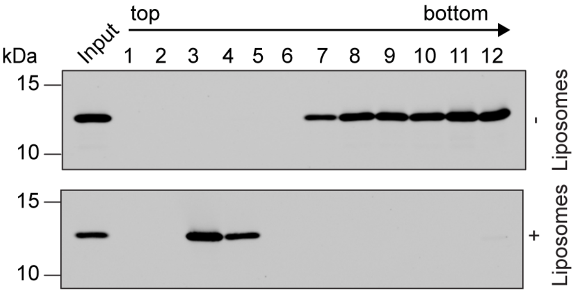

Dhiman *et al*., Supplementary Fig. 3

Supplementary Fig. 3:

Reconstitution of sUBXD8$_{71\text{-}132}$His into proteo-SUVs

Immunoblot analysis using anti-S-tag antibodies of fractions obtained after density gradient centrifugation to confirm efficient incorporation of sUBXD8$_{71\text{-}132}$His_S127C into SUVs. In the absence of liposomes, the protein remained in the high-density bottom fractions of the gradient. Upon reconstitution into SUVs, proteins were exclusively detected in the low-density fractions containing floating proteo-SUVs. Representative blot for 3 independent experiments.

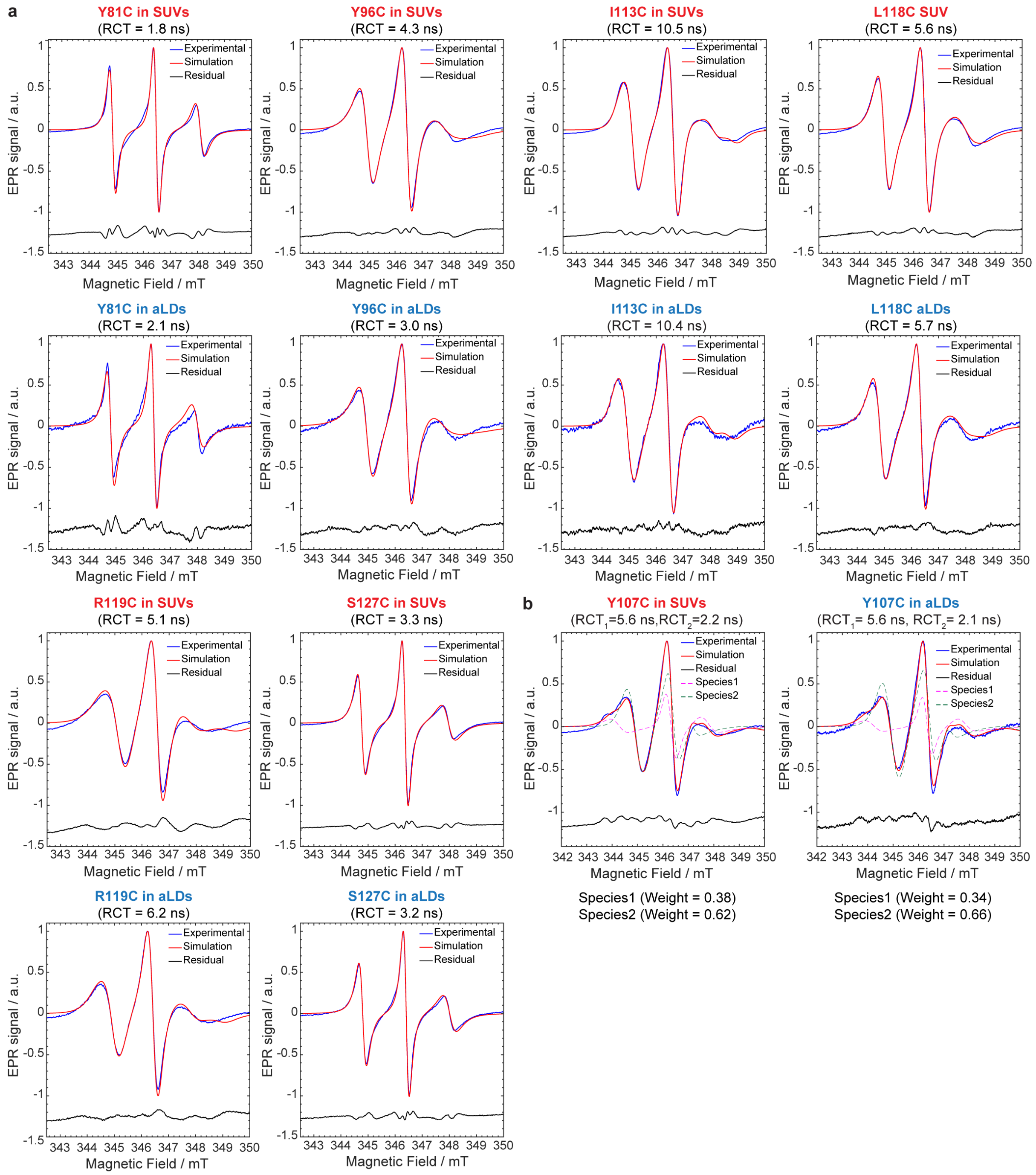

Dhiman *et al.*, Supplementary Fig. 4

Supplementary Fig. 4:

Simulation of X-band cwEPR spectra of MTSL spin-labeled sUBXD8$_{71\text{-}132}$His single cysteine mutants

(a) Experimental (blue) and simulated (red) X-band cwEPR spectra of MTSL spin-labeled sUBXD8$_{71\text{-}132}$His single cysteine mutants reconstituted into small unilamellar vesicles (SUVs) and into artificial LDs (aLDs) as indicated. The respective obtained rotational correlation time (RCT) values are shown in the title above each cwEPR spectrum. The residual line (black)

shows the difference between the experimental and simulated spectrum. Note that those sUBXD8$_{71\text{-}132}$His single cysteine mutants that showed two motional components in the low field region of the cwEPR spectra (marked with asterisks in Fig. 5e) were not utilized for these simulations. **(b)** Exemplary simulation of two motional components in MTSL spin-labeled sUBXD8$_{71\text{-}132}$His_Y107C reconstituted into SUVs (left) or into aLDs (right). Experimental (blue), simulated (red) and residuals (black) of X-band cwEPR spectra are indicated. The two motional components are implemented with dashed lines (species 1: magenta, species 2: green) and their respective weights are stated below the graphs. The simulation line (red) is the sum of both motional components with their respective weights. Source data are provided as a source data file.

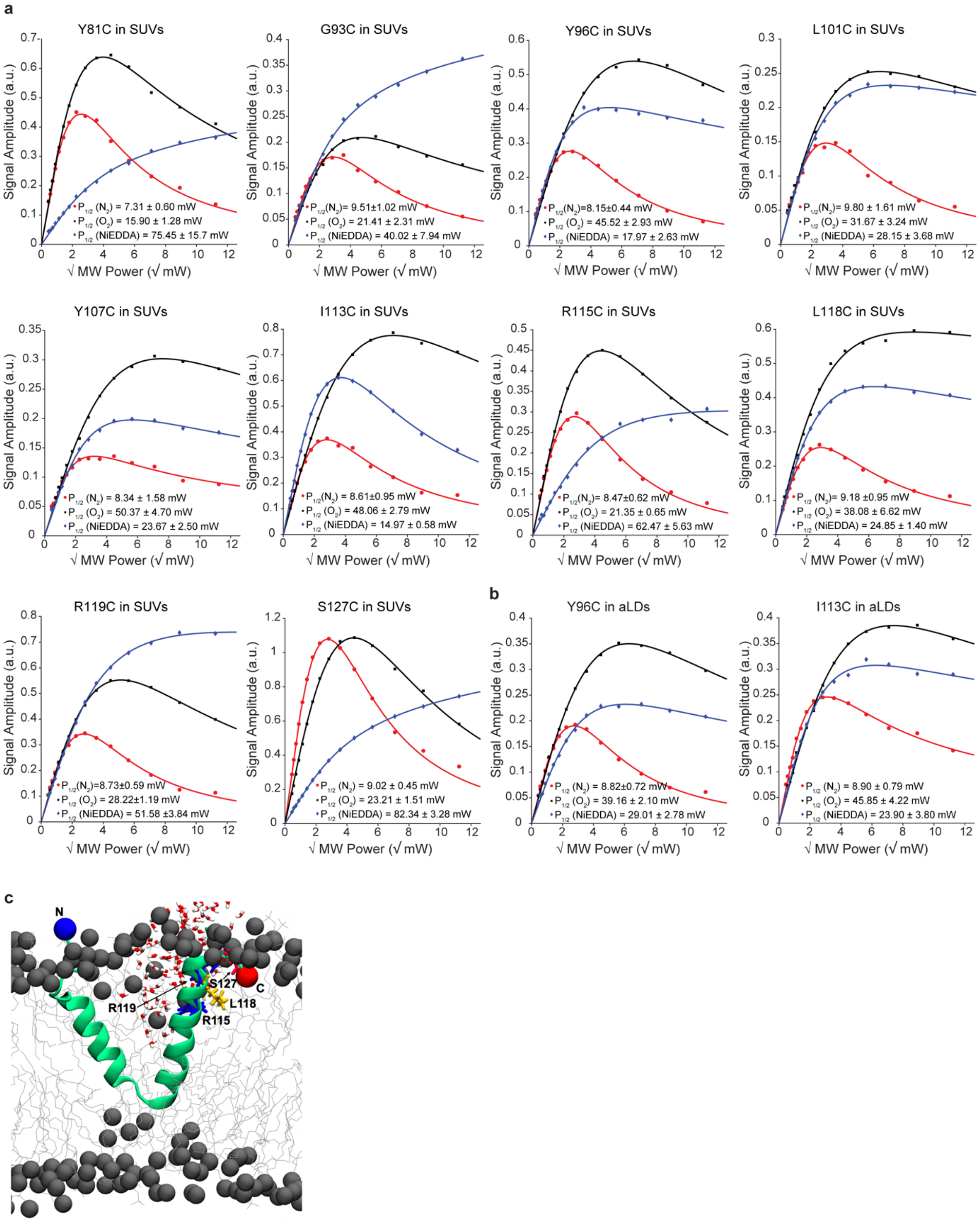

Dhiman *et al.*, Supplementary Fig. 5

Supplementary Fig. 5.

(a+b) EPR power saturation plots of MTSL spin-labeled sUBXD8$_{71\text{-}132}$His single cysteine mutants in SUVs (a) and aLDs (b). The peak-to-peak-amplitude of the central EPR line is plotted against the square root of the microwave power used. Power saturation curves were

measured under three conditions: nitrogen gas as control (red circles), molecular O_2 (black squares), and 50 mM NiEDDA (blue diamonds). $P_{1/2}$ values were obtained after curve fitting and are indicated with SEM. Data are representative for two independent experiments. **(c)** Average atomistic MD simulation structure of $UBXD8_{80-128}$ in a POPC bilayer as shown in Figure 3a, indicating that R115 is solvent-exposed in the membrane hydrophobic core. Water molecules are shown within 1 nm of residues R115, L118, R119 and S127. Source data are provided as a source data file.

figure 1c

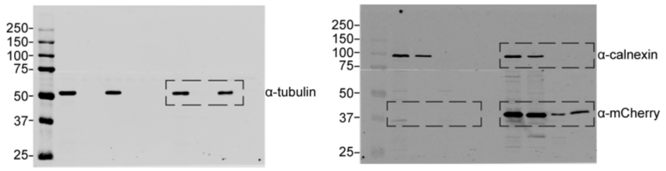

figure 1d

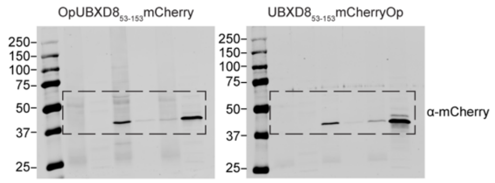

figure 1e

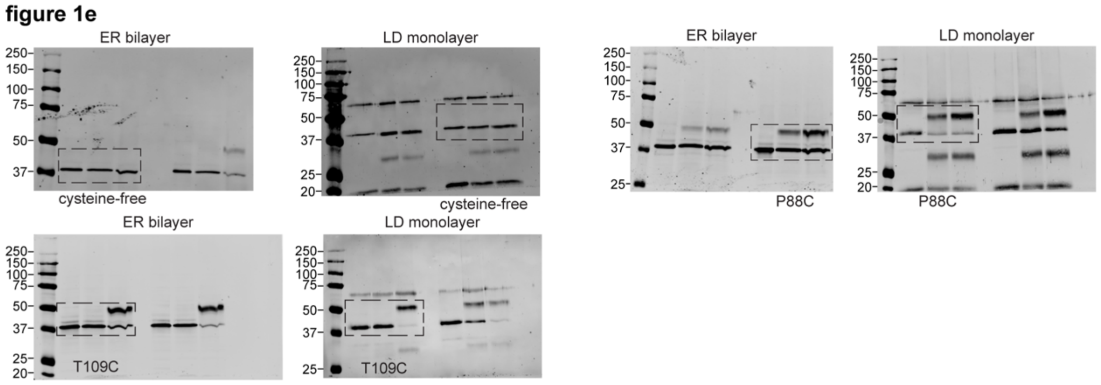

figure 2b

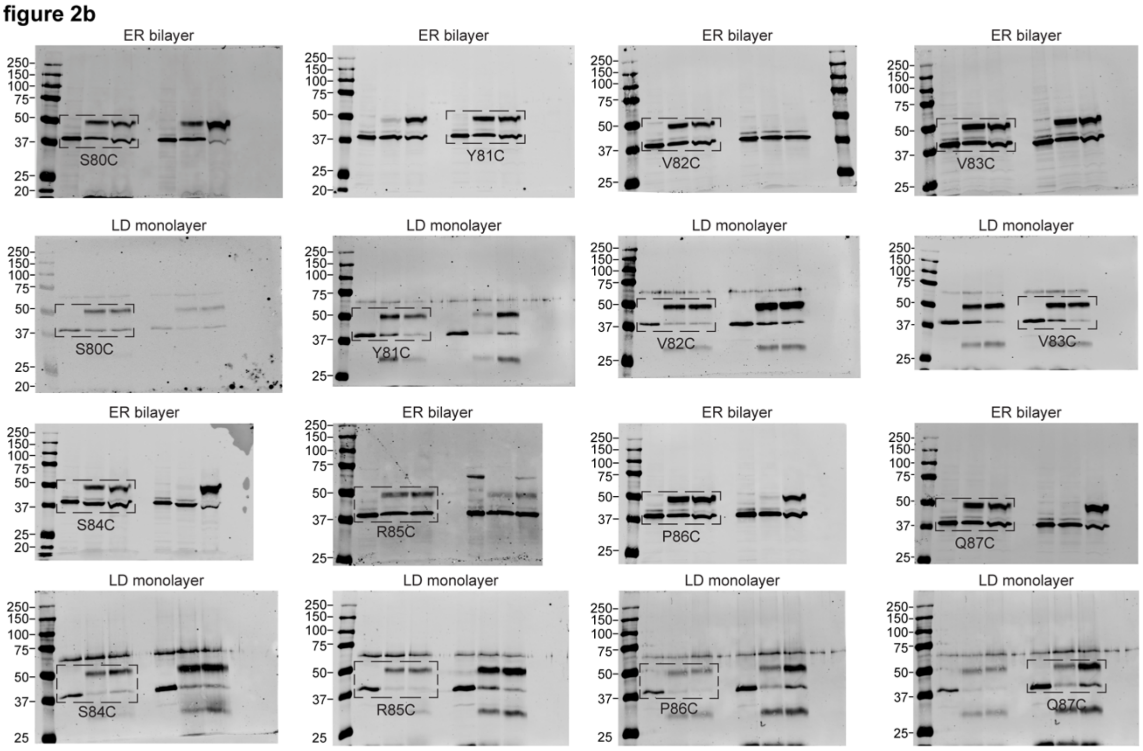

figure 2b continued

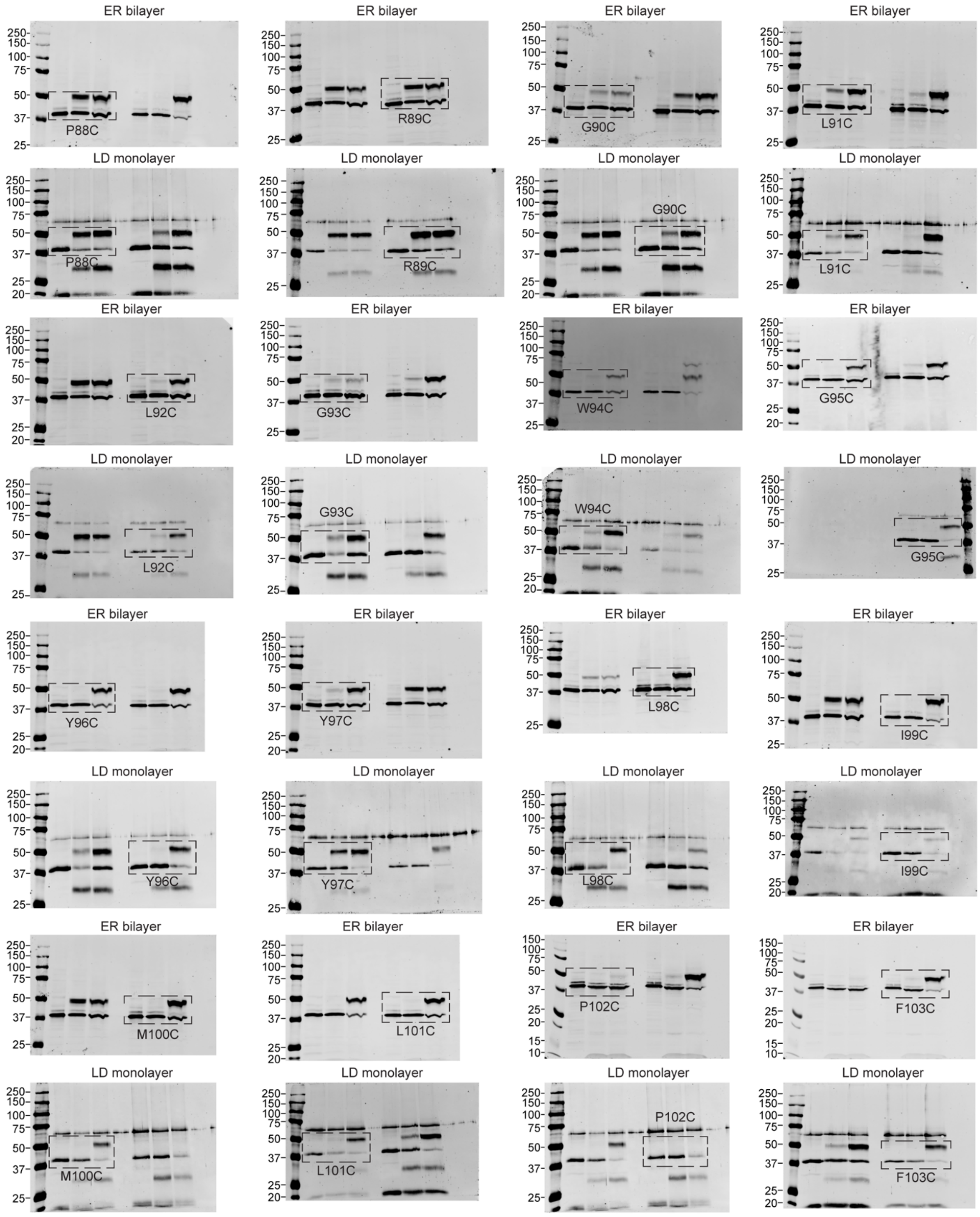

Dhiman *et al.*, Supplementary Fig. 6 continued

figure 2b continued

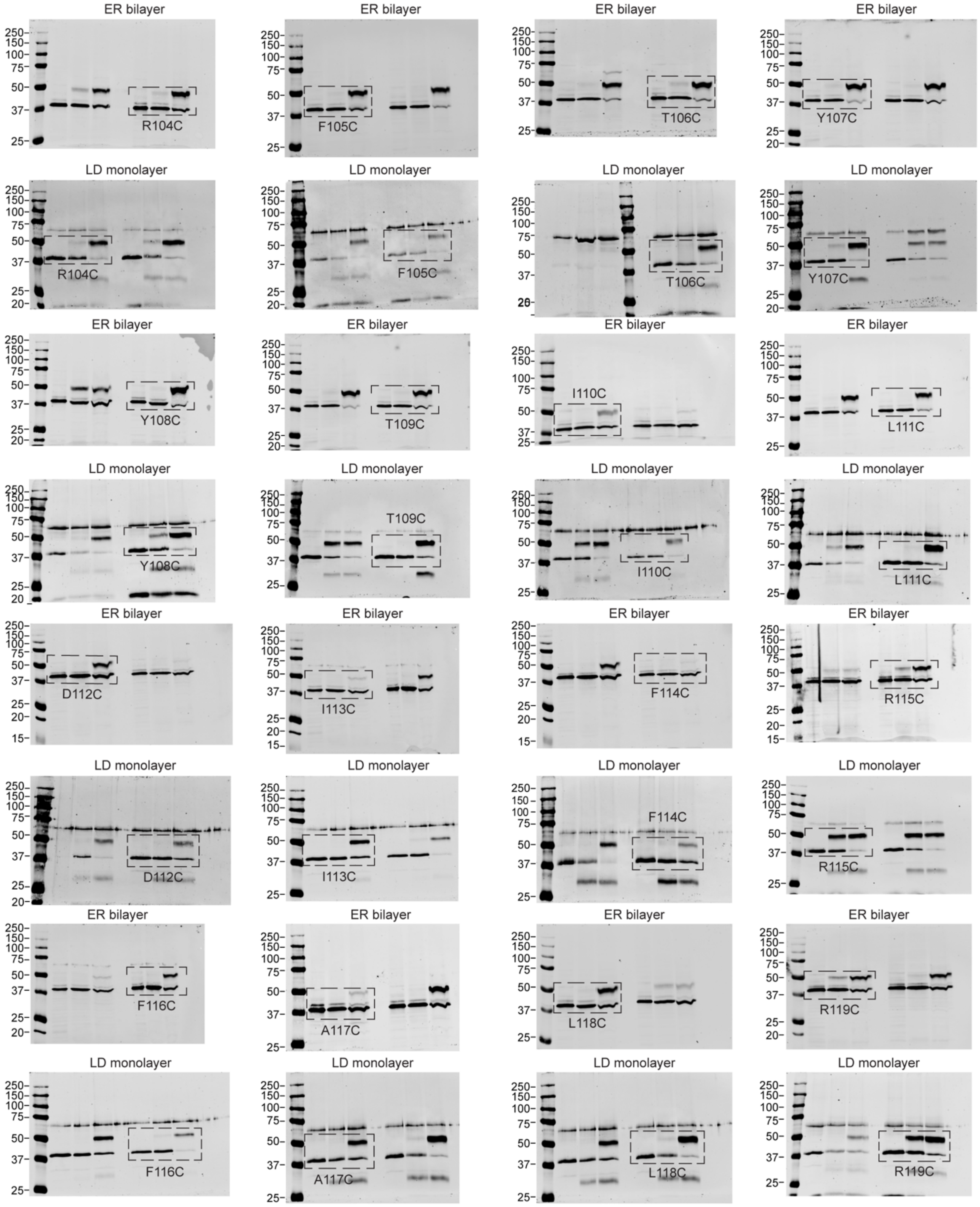

Dhiman *et al.*, Supplementary Fig. 6 continued

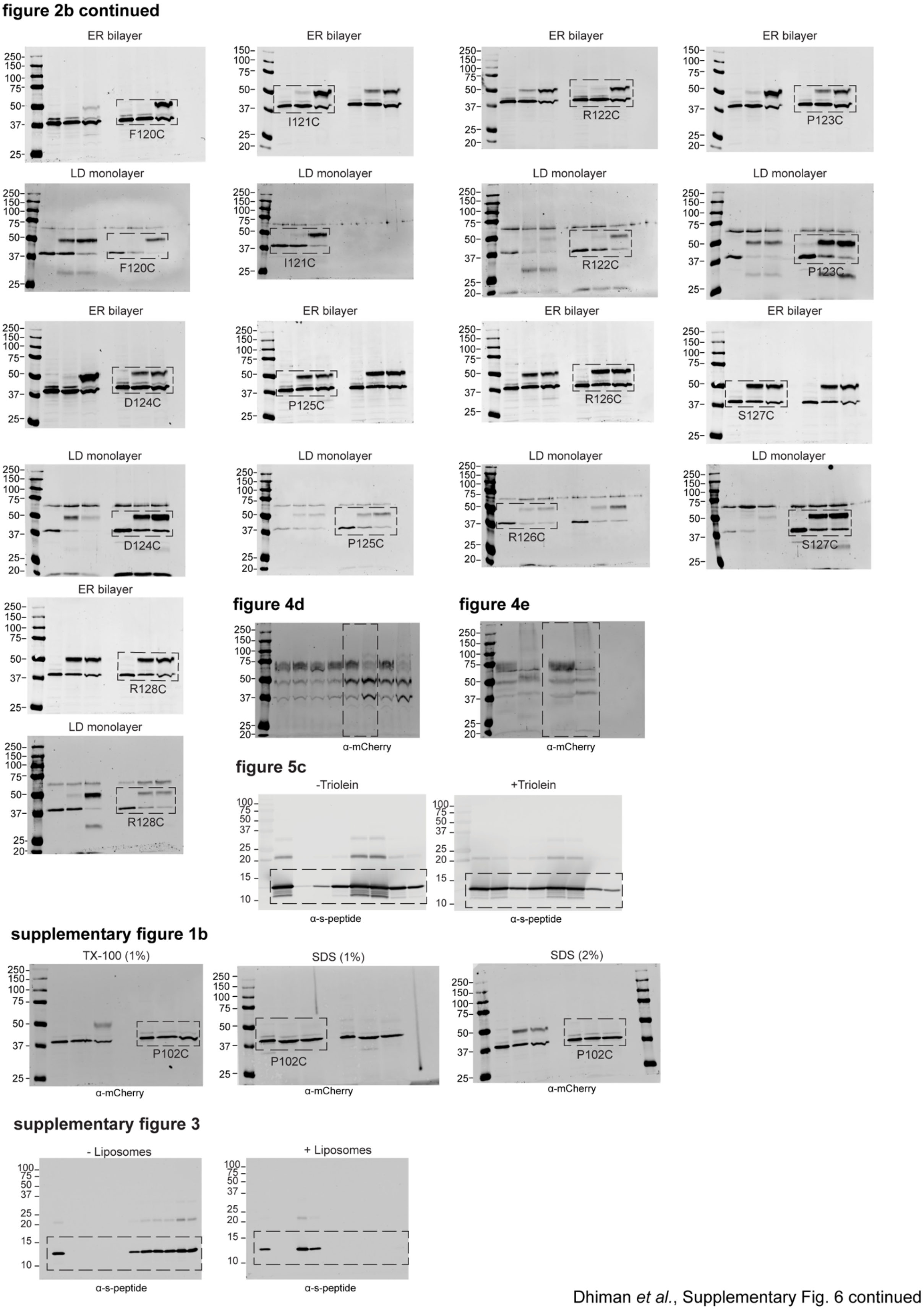

Supplementary Fig. 6. Uncropped immunoblots

Boxes indicate areas used for cropped panels.

Supplementary Table 1

Sequences of primers used for introducing single cysteines *via* site-directed mutagenesis and restriction-based cloning

**Primers**	**Sequence (5'-3')**
S80C_For	ctgaccacaggatctacTGCtatgttgtctcaagac
S80C_Rev	gtcttgagacaacataGCAgtagatcctgtggtcag
Y81C_For	ctacagcTGCgttgtctcaagacctcaaccaagg
Y81C_Rev	acaacGCAgctgtagatcctgtggtcagc
V82C_For	cagctatTGCgtctcaagacctcaaccaagggg
V82C_Rev	gagacGCAatagctgtagatcctgtggtcagc
V83C_For	ctatgttTGCtcaagacctcaaccaagggg
V83C_Rev	cttgaGCAaacatagctgtagatcctgtgg
S84C_For	tgttgtcTGCagacctcaaccaagggggc
S84C_Rev	ggtctGCAgacaacatagctgtagatcctgtgg
R85C_For	tgtctcaTGCcctcaaccaagggggctg
R85C_Rev	tgaggGCAtgagacaacatagctgtagatcctg
P86C_For	ctcaagaTGCcaaccaagggggctgcttg
P86C_Rev	ggttgGCAtcttgagacaacatagctgtagatc
Q87C_For	aagacctTGCccaagggggctgcttgga
Q87C_Rev	cttggGCAaggtcttgagacaacatagctgt
P88C_For	acctcaaTGCagggggctgcttggatgg
P88C_Rev	cccctGCAttgaggtcttgagacaacatagc
R89C_For	tcaaccaTGCgggctgcttggatggggt
R89C_Rev	agcccGCAtggttgaggtcttgagacaaca
G90C_For	accaaggTGCctgcttggatggggttattacttg
G90C_Rev	agcagGCAccttggttgaggtcttgagacaac
L91C_For	aagggggTGTcttggatggggttattacttgat
L91C_Rev	ccaagACAccccttggttgaggtct
L92C_For	ggggctgTGCggatggggttattacttgataatgc
L92C_Rev	ccccatccGCAcagccccttggttgagg
G93C_For	gctgcttTGCtggggttattacttgataatgcttc
G93C_Rev	ccccaGCAaagcagccccttggttg
W94C_For	gcttggaTGCggttattacttgataatgcttcc
W94C_Rev	taaccGCAtccaagcagccccttgg
G95C_For	tggatggTGCtattacttgataatgcttccattcc
G95C_Rev	agtaataGCAccatccaagcagcccct
Y96C_For	atggggtTGCtacttgataatgcttccattccgg
Y96C_Rev	aagtaGCAaccccatccaagcagccc
Y97C_For	gggttatTGCttgataatgcttccattccgg
Y97C_Rev	atcaaGCAataaccccatccaagcagcc
L98C_For	gctgcttggatggggttattacTGCataatgcttccattcc
L98C_Rev	ggaatggaagcattatGCAgtaataaccccatccaagcagc
I99C_For	ttacttgTGCatgcttccattccggtttacc
I99C_Rev	agcatGCAcaagtaataaccccatccaagcagc
M100C_For	cttgataTGTcttccattccggtttacctattac
M100C_Rev	ggaagACAtatcaagtaataaccccatccaagc
L101C_For	gataatgTGCccattccggtttacctattacacga

L101C_Rev	aatggGCAcattatcaagtaataaccccatcca
P102C_For	aatgcttTGCttccggtttacctattacacga
P102C_Rev	cggaaGCAaagcattatcaagtaataacccca
F103C_For	gcttccaTGCcggtttacctattacacgatacttg
F103C_Rev	aaccgGCAtggaagcattatcaagtaataaccc
R104C_For	tccattcTGTtttacctattacacgatacttgat
R104C_Rev	gtaaaACAgaatggaagcattatcaagt
F105C_For	attccggTGCacctattacacgatacttgatat
F105C_Rev	taggtGCAccggaatggaagcattatca
T106C_For	gataatgcttccattccggtttTGCtattacacgatacttg
T106C_Rev	caagtatcgtgtaataGCAaaaccggaatggaagcattatc
Y107C_For	gtttaccTGCtacacgatacttgatatatttaggt
Y107C_Rev	gtgtaGCAggtaaaccggaatggaagca
Y108C_For	tacctatTGCacgatacttgatatatttaggtttg
Y108C_Rev	atcgtGCAataggtaaaccggaatggaag
T109C_For	ctattacTGCatacttgatatatttaggtttgctc
T109C_Rev	agtatGCAgtaataggtaaaccggaatgg
I110C_For	ttacacgTGCcttgatatatttaggtttgctcttc
I110C_Rev	tcaagGCAcgtgtaataggtaaaccggaatg
L111C_For	cacgataTGCgatatatttaggtttgctcttcgtt
L111C_Rev	atatcGCAtatcgtgtaataggtaaaccggaa
D112C_For	gatacttTGCatatttaggtttgctcttcgtttt
D112C_Rev	aatatGCAaagtatcgtgtaataggtaaaccgg
I113C_For	acttgatTGCtttaggtttgctcttcgttttatac
I113C_Rev	ctaaaGCAatcaagtatcgtgtaataggtaaac
F114C_For	tgatataTGCaggtttgctcttcgttttatac
F114C_Rev	aacctGCAtatatcaagtatcgtgtaataggt
R115C_For	tatatttTGCtttgctcttcgttttatac
R115C_Rev	gcaaaGCAaaatatatcaagtatcgtgtaatag
F116C_For	atttaggTGCgctcttcgttttatacggcctga
F116C_Rev	agagcGCAcctaaatatatcaagtatcgtgtaa
A117C_For	taggtttTGTcttcgttttatacggcctgacc
A117C_Rev	cgaagACAaaacctaaatatatcaagtatcgtg
L118C_For	gtttgctTGCcgttttatacggcctgacc
L118C_Rev	aaacgGCAagcaaacctaaatatatcaagtatc
R119C_For	tgctcttTGCtttatacggcctgaccctc
R119C_Rev	ataaaGCAaagagcaaacctaaatatatcaag
F120C_For	tcttcgtTGCatacggcctgaccctcgc
F120C_Rev	cgtatGCAacgaagagcaaacctaaatatatca
I121C_For	tcgttttTGCcggcctgaccctcgcagc
I121C_Rev	ggccgGCAaaaacgaagagcaaacct
R122C_For	ttttataTGCcctgaccctcgcagccgg
R122C_Rev	tcaggGCAtataaaacgaagagcaaacc
P123C_For	tatacggTGCgaccctcgcagccgggtc
P123C_Rev	gggtcGCAccgtataaaacgaagagcaaacc
D124C_For	acggcctTGCcctcgcagccgggtcactg
D124C_Rev	cgaggGCAaggccgtataaaacgaagagcaaac
P125C_For	gcctgacTGCcgcagccgggtcactgac

P125C_Rev	ctgcgGCAgtcaggccgtataaaacgaagagc
R126C_For	tgaccctTGCagccgggtcactgacccc
R126C_Rev	cggctGCAagggtcaggccgtataaaacg
S127C_For	ccctcgcTGCcgggtcactgaccccgtt
S127C_Rev	acccgGCAgcgagggtcaggccgtat
R128C_For	tcgcagcTGCgtcactgaccccgttgggg
R128C_Rev	gtgacGCAgctgcgagggtcaggccg
mCherry_EcoRI_For	agtactGAATTCatggtgagcaagggcgaggag
mCherry_KpnI_Rev	gcatGGTACCttacttgtacagctcgtccatg
mCherry_Op_KpnI_Rev	agtacGGTACCtaaGCCCGTCTTGTTGGAGAAAGG CACGTAGAAGTTTGGGCCCTTGTACAGCTCGTC CATgcc
OpUBXD8$_{53-153}$XbaI_For	acTCTAGAgccaccATGGGCCCAAACTTCTACGT GCCTTTCTCCAACAAGACGGGCgagcaagagggcgt acctag
UBXD8$_{53-153}$EcoRI_Rev	tagctGAATTCgacagggtgtgccctccc
UBXD8$_{53-153}$XbaI_For	acTCTAGAgccaccatggagcaagagggcgtacctag
UBXD8$_{71-132}$BamHI_For	gcgcGGATCCgttaatacagctgaccac
UBXD8$_{71_132}$T130C_NotI _Rev	gcgcGCGGCCGCtcagtgatggtgatggtgatgggggtcACAgacccggc tgcgaggg
UBXD8$_{71_132}$NotI_Rev	gcgcGCGGCCGCtcagtgatggtgatggtgatgggggtcagtgacccggctg cgaggg
UBXD8$_{71_132}$S127C_NotI_ Rev	gcgcGCGGCCGCtcagtgatggtgatggtgatgggggtcagtgacccggca gcgaggg

Supplementary Table 2
List of plasmids generated in this study

**Plasmid name**	**Cloning strategy**	**Vector backbone**
Op_UBXD8_$_{53-153}$_S80C_mCherry	Site-directed mutagenesis	pCDNA 3.1(-)
Op_UBXD8_$_{53-153}$_Y81C_mCherry	Site-directed mutagenesis	pCDNA 3.1(-)
Op_UBXD8_$_{53-153}$_V82C_mCherry	Site-directed mutagenesis	pCDNA 3.1(-)
Op_UBXD8_$_{53-153}$_V83C_mCherry	Site-directed mutagenesis	pCDNA 3.1(-)
Op_UBXD8_$_{53-153}$_S84C_mCherry	Site-directed mutagenesis	pCDNA 3.1(-)
Op_UBXD8_$_{53-153}$_R85C_mCherry	Site-directed mutagenesis	pCDNA 3.1(-)
Op_UBXD8_$_{53-153}$_P86C_mCherry	Site-directed mutagenesis	pCDNA 3.1(-)
Op_UBXD8_$_{53-153}$_Q87C_mCherry	Site-directed mutagenesis	pCDNA 3.1(-)
Op_UBXD8_$_{53-153}$_P88C_mCherry	Site-directed mutagenesis	pCDNA 3.1(-)
Op_UBXD8_$_{53-153}$_R89C_mCherry	Site-directed mutagenesis	pCDNA 3.1(-)
Op_UBXD8_$_{53-153}$_G90C_mCherry	Site-directed mutagenesis	pCDNA 3.1(-)
Op_UBXD8_$_{53-153}$_L91C_mCherry	Site-directed mutagenesis	pCDNA 3.1(-)
Op_UBXD8_$_{53-153}$_L92C_mCherry	Site-directed mutagenesis	pCDNA 3.1(-)
Op_UBXD8_$_{53-153}$_G93C_mCherry	Site-directed mutagenesis	pCDNA 3.1(-)
Op_UBXD8_$_{53-153}$_W94C_mCherry	Site-directed mutagenesis	pCDNA 3.1(-)
Op_UBXD8_$_{53-153}$_G95C_mCherry	Site-directed mutagenesis	pCDNA 3.1(-)
Op_UBXD8_$_{53-153}$_Y96C_mCherry	Site-directed mutagenesis	pCDNA 3.1(-)
Op_UBXD8_$_{53-153}$_Y97C_mCherry	Site-directed mutagenesis	pCDNA 3.1(-)

Op_UBXD8_$_{53-153}$_L98C_mCherry	Site-directed mutagenesis	pCDNA 3.1(-)
Op_UBXD8_$_{53-153}$_I99C_mCherry	Site-directed mutagenesis	pCDNA 3.1(-)
Op_UBXD8_$_{53-153}$_M100C_mCherry	Site-directed mutagenesis	pCDNA 3.1(-)
Op_UBXD8_$_{53-153}$_L101C_mCherry	Site-directed mutagenesis	pCDNA 3.1(-)
Op_UBXD8_$_{53-153}$_P102C_mCherry	Site-directed mutagenesis	pCDNA 3.1(-)
Op_UBXD8_$_{53-153}$_F103C_mCherry	Site-directed mutagenesis	pCDNA 3.1(-)
Op_UBXD8_$_{53-153}$_R104C_mCherry	Site-directed mutagenesis	pCDNA 3.1(-)
Op_UBXD8_$_{53-153}$_F105C_mCherry	Site-directed mutagenesis	pCDNA 3.1(-)
Op_UBXD8_$_{53-153}$_T106C_mCherry	Site-directed mutagenesis	pCDNA 3.1(-)
Op_UBXD8_$_{53-153}$_Y107C_mCherry	Site-directed mutagenesis	pCDNA 3.1(-)
Op_UBXD8_$_{53-153}$_Y108C_mCherry	Site-directed mutagenesis	pCDNA 3.1(-)
Op_UBXD8_$_{53-153}$_T109C_mCherry	Site-directed mutagenesis	pCDNA 3.1(-)
Op_UBXD8_$_{53-153}$_I110C_mCherry	Site-directed mutagenesis	pCDNA 3.1(-)
Op_UBXD8_$_{53-153}$_L111C_mCherry	Site-directed mutagenesis	pCDNA 3.1(-)
Op_UBXD8_$_{53-153}$_D112C_mCherry	Site-directed mutagenesis	pCDNA 3.1(-)
Op_UBXD8_$_{53-153}$_I113C_mCherry	Site-directed mutagenesis	pCDNA 3.1(-)
Op_UBXD8_$_{53-153}$_F114C_mCherry	Site-directed mutagenesis	pCDNA 3.1(-)
Op_UBXD8_$_{53-153}$_R115C_mCherry	Site-directed mutagenesis	pCDNA 3.1(-)
Op_UBXD8_$_{53-153}$_F116C_mCherry	Site-directed mutagenesis	pCDNA 3.1(-)
Op_UBXD8_$_{53-153}$_A117C_mCherry	Site-directed mutagenesis	pCDNA 3.1(-)
Op_UBXD8_$_{53-153}$_L118C_mCherry	Site-directed mutagenesis	pCDNA 3.1(-)
Op_UBXD8_$_{53-153}$_R119C_mCherry	Site-directed mutagenesis	pCDNA 3.1(-)
Op_UBXD8_$_{53-153}$_F120C_mCherry	Site-directed mutagenesis	pCDNA 3.1(-)
Op_UBXD8_$_{53-153}$_I121C_mCherry	Site-directed mutagenesis	pCDNA 3.1(-)
Op_UBXD8_$_{53-153}$_R122C_mCherry	Site-directed mutagenesis	pCDNA 3.1(-)
Op_UBXD8_$_{53-153}$_P123C_mCherry	Site-directed mutagenesis	pCDNA 3.1(-)
Op_UBXD8_$_{53-153}$_D124C_mCherry	Site-directed mutagenesis	pCDNA 3.1(-)
Op_UBXD8_$_{53-153}$_P125C_mCherry	Site-directed mutagenesis	pCDNA 3.1(-)
Op_UBXD8_$_{53-153}$_R126C_mCherry	Site-directed mutagenesis	pCDNA 3.1(-)
Op_UBXD8_$_{53-153}$_S127C_mCherry	Site-directed mutagenesis	pCDNA 3.1(-)
Op_UBXD8_$_{53-153}$_R128C_mCherry	Site-directed mutagenesis	pCDNA 3.1(-)
Op_UBXD8_$_{53-153}$_L91C_L118C_mCherry	Site-directed mutagenesis	pCDNA 3.1(-)
Op_UBXD8_$_{53-153}$_mCherry	Restriction-based cloning	pCDNA 3.1(-)
UBXD8_$_{53-153}$_mCherry_Op	Restriction-based cloning	pCDNA 3.1(-)
GST_PP_S_UBXD8$_{71-132}$_Y81C_6His	Restriction-based cloning	pGEX6P
GST_PP_S_UBXD8$_{71-132}$_G93C_6His	Restriction-based cloning	pGEX6P
GST_PP_S_UBXD8$_{71-132}$_Y96C_6His	Restriction-based cloning	pGEX6P
GST_PP_S_UBXD8$_{71-132}$_L101C_6His	Restriction-based cloning	pGEX6P
GST_PP_S_UBXD8$_{71-132}$_Y107C_6His	Restriction-based cloning	pGEX6P
GST_PP_S_UBXD8$_{71-132}$_I113C_6His	Restriction-based cloning	pGEX6P
GST_PP_S_UBXD8$_{71-132}$_R115C_6His	Restriction-based cloning	pGEX6P
GST_PP_S_UBXD8$_{71-132}$_L118C_6His	Restriction-based cloning	pGEX6P
GST_PP_S_UBXD8$_{71-132}$_R119C_6His	Restriction-based cloning	pGEX6P
GST_PP_S_UBXD8$_{71-132}$_S127C_6His	Restriction-based cloning	pGEX6P
GST_PP_S_UBXD8$_{71-132}$_T130C_6His	Restriction-based cloning	pGEX6P
