# Supplementary material for: Hairpin protein partitioning from the ER to lipid droplets involves major structural rearrangements
Source: Nat Commun. 2024 May 27;15:4504. doi: 10.1038/s41467-024-48843-8 (PMC11130287; doi:10.1038/s41467-024-48843-8)
Supplement: Supplementary file 3 — Reporting Summary [file 41467_2024_48843_MOESM3_ESM.pdf]

nature portfolio

Corresponding author(s): Bianca Schrul

Last updated by author(s): May 2, 2024

Reporting Summary

Nature Portfolio wishes to improve the reproducibility of the work that we publish. This form provides structure for consistency and transparency in reporting. For further information on Nature Portfolio policies, see our Editorial Policies and the Editorial Policy Checklist.

Statistics

For all statistical analyses, confirm that the following items are present in the figure legend, table legend, main text, or Methods section.

n/a	Confirmed	
☐	☒	The exact sample size (*n*) for each experimental group/condition, given as a discrete number and unit of measurement
☐	☒	A statement on whether measurements were taken from distinct samples or whether the same sample was measured repeatedly
☒	☐	The statistical test(s) used AND whether they are one- or two-sided<br>*Only common tests should be described solely by name; describe more complex techniques in the Methods section.*
☒	☐	A description of all covariates tested
☒	☐	A description of any assumptions or corrections, such as tests of normality and adjustment for multiple comparisons
☐	☒	A full description of the statistical parameters including central tendency (e.g. means) or other basic estimates (e.g. regression coefficient) AND variation (e.g. standard deviation) or associated estimates of uncertainty (e.g. confidence intervals)
☒	☐	For null hypothesis testing, the test statistic (e.g. *F*, *t*, *r*) with confidence intervals, effect sizes, degrees of freedom and *P* value noted<br>*Give P values as exact values whenever suitable.*
☒	☐	For Bayesian analysis, information on the choice of priors and Markov chain Monte Carlo settings
☒	☐	For hierarchical and complex designs, identification of the appropriate level for tests and full reporting of outcomes
☒	☐	Estimates of effect sizes (e.g. Cohen's *d*, Pearson's *r*), indicating how they were calculated

Our web collection on statistics for biologists contains articles on many of the points above.

Software and code

Policy information about availability of computer code

Data collection	All MD simulations were carried out using publicly available open source code GROMACS, versions 2020.2 and 2021.1
Data analysis	Analyses were performed using inbuilt GROMACS 2020.2, 2021 tools, VMD 1.9.4, Easyspin version 5.2.35 and Matlab Version 23.4 (R2023b). Data were plotted using matplotlib. Matlab protocols used for analyzing cwEPR data were deposited at Zenodo.org (https://doi.org/10.5281/zenodo.11036547). The files will be made available to the public upon acceptance of the manuscript.

For manuscripts utilizing custom algorithms or software that are central to the research but not yet described in published literature, software must be made available to editors and reviewers. We strongly encourage code deposition in a community repository (e.g. GitHub). See the Nature Portfolio guidelines for submitting code & software for further information.

Data

Policy information about availability of data

All manuscripts must include a data availability statement. This statement should provide the following information, where applicable:

- Accession codes, unique identifiers, or web links for publicly available datasets
- A description of any restrictions on data availability
- For clinical datasets or third party data, please ensure that the statement adheres to our policy

Datasets generated during this study are available in the manuscript and/or in the supplementary information. Source data are provided with the manuscript.

Original cwEPR datasets and MD simulation systems (structures, topologies, MD parameter and force field files) were deposited at Zenodo.org (https://doi.org/10.5281/zenodo.11036547). The files will be made available to the public upon acceptance of the manuscript.

Research involving human participants, their data, or biological material

Policy information about studies with human participants or human data. See also policy information about sex, gender (identity/presentation), and sexual orientation and race, ethnicity and racism.

Reporting on sex and gender	No human participants were involved in this study.
Reporting on race, ethnicity, or other socially relevant groupings	No human participants were involved in this study.
Population characteristics	No human participants were involved in this study.
Recruitment	No human participants were involved in this study.
Ethics oversight	No human participants were involved in this study.

Note that full information on the approval of the study protocol must also be provided in the manuscript.

Field-specific reporting

Please select the one below that is the best fit for your research. If you are not sure, read the appropriate sections before making your selection.

- [x] Life sciences
- [ ] Behavioural & social sciences
- [ ] Ecological, evolutionary & environmental sciences

For a reference copy of the document with all sections, see nature.com/documents/nr-reporting-summary-flat.pdf

Life sciences study design

All studies must disclose on these points even when the disclosure is negative.

Sample size	Simulation of membrane patches, simulation time and replication were large enough to exclude any periodic boundary artifacts or sampling issues or reproducibility issues.
Data exclusions	No data were excluded from the analyses.
Replication	All data in this manuscript have been successfully reproduced as outlined in the respective figure legends and/or the methods section. All attempts of replication simulation results were successful.
Randomization	Sample randomization is not relevant to this study as no populations were investigated. Randomization would not have changed the results.
Blinding	Blinding was not relevant to this study and would not have changed the results.

Reporting for specific materials, systems and methods

We require information from authors about some types of materials, experimental systems and methods used in many studies. Here, indicate whether each material, system or method listed is relevant to your study. If you are not sure if a list item applies to your research, read the appropriate section before selecting a response.

Materials & experimental systems

n/a	Involved in the study	
☐	☒	Antibodies
☐	☒	Eukaryotic cell lines
☒	☐	Palaeontology and archaeology
☒	☐	Animals and other organisms
☒	☐	Clinical data
☒	☐	Dual use research of concern
☒	☐	Plants

Methods

n/a	Involved in the study	
☒	☐	ChIP-seq
☒	☐	Flow cytometry
☒	☐	MRI-based neuroimaging

Antibodies

Antibodies used

Primary antibodies:
rabbit anti-mCherry (Invitrogen, PA534974, 1:5,000),
rabbit anti-calnexin (Enzo Life Sciences, ADI-SPA-865-F, 1:2,000),
mouse anti-tubulin (Sigma-Aldrich, T6199-100UL,1:10,000),
rabbit anti-his-tag (Cell Signaling, 2365S, 1:2,000)
mouse anti-S-peptide (Invitrogen, MA1-981,1:3,000).
IRDye-conjugated secondary antibodies against rabbit (680LT, 926-68021, 1:20,000) and mouse (800CW, 926-32210, 1:15,000) were obtained from Li-Cor Biosciences.

Validation

All antibodies were validated by the respective manufacturers.

Eukaryotic cell lines

Policy information about cell lines and Sex and Gender in Research

Cell line source(s)

HeLa Kyoto cells were provided by Ron Kopito (Stanford University, CA, USA). (PMID: 27295553)

Authentication

Cell lines were not authenticated

Mycoplasma contamination

Cells were routinely tested for the absence of mycoplasma.

Commonly misidentified lines
(See ICLAC register)

none

Plants

Seed stocks

Report on the source of all seed stocks or other plant material used. If applicable, state the seed stock centre and catalogue number. If plant specimens were collected from the field, describe the collection location, date and sampling procedures.

Novel plant genotypes

Describe the methods by which all novel plant genotypes were produced. This includes those generated by transgenic approaches, gene editing, chemical/radiation-based mutagenesis and hybridization. For transgenic lines, describe the transformation method, the number of independent lines analyzed and the generation upon which experiments were performed. For gene-edited lines, describe the editor used, the endogenous sequence targeted for editing, the targeting guide RNA sequence (if applicable) and how the editor was applied.

Authentication

Describe any authentication procedures for each seed stock used or novel genotype generated. Describe any experiments used to assess the effect of a mutation and, where applicable, how potential secondary effects (e.g. second site T-DNA insertions, mosiacism, off-target gene editing) were examined.
